# The fuzzy system ensembles entomological, epidemiological, demographic and environmental data to unravel the dengue transmission risk in an endemic city

**DOI:** 10.1186/s12889-024-19942-4

**Published:** 2024-09-27

**Authors:** André de Souza Leandro, Felipe de Oliveira, Renata Defante Lopes, Açucena Veleh Rivas, Caroline Amaral Martins, Isaac Silva, Daniel A. M. Villela, Marcello Goulart Teixeira, Samanta Cristina das Chagas Xavier, Rafael Maciel-de-Freitas

**Affiliations:** 1 Centro de Controle de Zoonoses da Secretaria Municipal de Saúde de Foz do Iguaçu,, Foz do Iguaçu, PR Brazil; 2 Laboratório de Mosquitos Transmissores de Hematozoários, Instituto Oswaldo Cruz, Fiocruz - IOC, Rio de Janeiro, RJ Brazil; 3 Laboratório de Biologia de Tripanosomatídeos, Instituto Oswaldo Cruz - IOC, Rio de Janeiro, RJ Brazil; 4 Universidade Federal Latino-Americana, Foz do Iguaçu, PR Brazil; 5 Fundação Itaiguapy, Instituto de Ensino e Pesquisa, Laboratório de Saúde Única do Centro de Medicina Tropical da Tríplice Fronteira,, Foz do Iguaçu, PR Brazil; 6 Departamento de Ciências Patológicas, Universidade Estadual de Londrina, Londrina, PR Brazil; 7 Programa de Computação Científica, Fundação Oswaldo Cruz, Rio de Janeiro, RJ Brazil; 8 Instituto de Computação, Universidade Federal do Rio de Janeiro, Rio de Janeiro, RJ Brazil; 9 Bernhard Nocht Institute for Tropical Medicine, Hamburg, Germany

**Keywords:** *Aedes*, Surveillance, Arboviruses, Vector control, Hotspots

## Abstract

**Background:**

The effectiveness of dengue control interventions depends on an effective integrated surveillance system that involves analysis of multiple variables associated with the natural history and transmission dynamics of this arbovirus. Entomological indicators associated with other biotic and abiotic parameters can assertively characterize the spatiotemporal trends related to dengue transmission risk. However, the unpredictability of the non-linear nature of the data, as well as the uncertainty and subjectivity inherent in biological data are often neglected in conventional models.

**Methods:**

As an alternative for analyzing dengue-related data, we devised a fuzzy-logic approach to test ensembles of these indicators across categories, which align with the concept of degrees of truth to characterize the success of dengue transmission by *Aedes aegypti* mosquitoes in an endemic city in Brazil. We used locally gathered entomological, demographic, environmental and epidemiological data as input sources using freely available data on digital platforms. The outcome variable, risk of transmission, was aggregated into three categories: low, medium, and high. Spatial data was georeferenced and the defuzzified values were interpolated to create a map, translating our findings to local public health managers and decision-makers to direct further vector control interventions.

**Results:**

The classification of low, medium, and high transmission risk areas followed a seasonal trend expected for dengue occurrence in the region. The fuzzy approach captured the 2020 outbreak, when only 14.06% of the areas were classified as low risk. The classification of transmission risk based on the fuzzy system revealed effective in predicting an increase in dengue transmission, since more than 75% of high-risk areas had an increase in dengue incidence within the following 15 days.

**Conclusions:**

Our study demonstrated the ability of fuzzy logic to characterize the city’s spatiotemporal heterogeneity in relation to areas at high risk of dengue transmission, suggesting it can be considered as part of an integrated surveillance system to support timely decision-making.

**Supplementary Information:**

The online version contains supplementary material available at 10.1186/s12889-024-19942-4.

## Introduction

Among the diverse arboviruses, some pose significant concerns due to their pathogenic behavior and the potential to cause severe outbreaks in densely populated urban areas. The globally distributed *Aedes aegypti* mosquito is identified as the primary vector for arboviruses such as dengue (DENV), Zika (ZIKV), and chikungunya (CHIKV). This mosquito species lives in close association with human dwellings, preferring to feed on human blood. It lays its eggs in man-made containers situated in the vicinity of human houses and has a limited flight range from its breeding site [1–5]. Consequently, understanding the biology of *Ae. aegypti* in endemic field settings is crucial for comprehending disease epidemiology and designing more effective control strategies. [6]. Among the arboviruses, dengue stands out for being the most significant disease caused by arboviruses on the planet. It is rapidly expanding globally, with a record increase in incidence in recent years, where half the world’s population is at risk of infection due to climatic and environmental conditions that favor the proliferation of the main vector, *Aedes aegypti*, as well as demographic and immunological factors of the host. Dengue is caused by four genetically distinct serotypes of the DENV virus (DENV-1, -2, -3 and − 4), and is related to high global morbidity and mortality rates. DENV is actually endemic in more than 100 countries, causes major economic losses and deaths due to its most severe form [7, 8]. The effectiveness of dengue control actions depends on a robust surveillance system. Entomological surveillance practices, based on systematic collection of samples from mosquito populations in the field, need to be improved, but are fundamental to the evidence-based decision-making process [9]. Ideally, mosquito counts should be analyzed spatially and temporally to create maps that translate this information into spatiotemporal transmission risk models. The next step would be to identify high-risk regions within a city for disease transmission [10, 11]. This approach enables targeted vector control interventions, fostering a more effective integrated strategy of surveillance and mosquito control. Effective surveillance guides risk assessment and response to epidemics, facilitating disease trend monitoring and program evaluation [12, 13].

Epidemiological data collection, both active and passive, is the most widely used form of surveillance and can be complemented by entomological, socioeconomic, and environmental data. Passive surveillance of epidemiological data relies on disease notifications from health units, which is often underestimated [9, 14]. Thus, implementing epidemiological analysis of notified data, syndromic surveillance, laboratory notification, and active surveillance strategies is recommended [13, 15, 16]. Processing, sharing, and timely analysis of epidemiological data in metropolitan endemic sites pose challenges [17, 18]. Modern technologies are essential for facilitating notification and enabling more effective dissemination, analysis, and data integration for decision-making, adhering to the principles of precision epidemiology, fundamental for success in health actions [19–21].

To understand epidemiological patterns in a disease outbreak, computational tools and mathematical models are essential for demonstrating cause-and-effect relationships and evaluating evidence for infectious disease certainty. However, prevention strategies may be limited due to the complex and dynamic nature of the disease, including environmental factors, vectors, and hosts, especially dengue transmission dynamics. Public health managers must consolidate data from various sources for an integrated approach [22, 23]. The unpredictability in the nonlinear nature of data, uncertainty, and subjectivity inherent in biological data are often neglected in conventional models. Still, these challenges can be addressed with appropriate mathematical tools, such as fuzzy logic [24, 25].

Environmental and biological phenomena exhibit continuous and gradual transitions. Unlike classical logic models, which use discrete units with clear limits, fuzzy logic models can represent the spatial distribution of continuous biological phenomena more realistically [24, 25]. Fuzzy logic mimics human thinking processes, enhancing decisions by considering imprecision and intermediate values between 0 and 1, which align with the concept of degrees of truth. This contrasts with the binary focus of classical logic models, which demand high precision with only dual interpretations like 0 or 1, false or true [25–27].

In this context, we present an example of applying fuzzy logic to characterize the successful transmission of dengue by *Ae. aegypti* in an endemic city in Brazil. We utilized locally gathered entomological, demographic, environmental, and epidemiological data as input sources. The output variable, transmission risk, was aggregated into three categories: low, medium, and high. We then translated the fuzzy logic into a map, facilitating the identification of areas with varying transmission risks. The study aimed to assess whether fuzzy logic effectively indicates high-risk areas for dengue outbreaks in Foz do Iguaçu from 2017 to 2022. This work proposes using a fuzzy logic-based system to evaluate the receptivity of areas for dengue transmission, serving as a decision-making tool to prioritize areas for intervention and mitigate dengue transmission by *Ae. aegypti* in urban settlements.

## Materials and methods

### Study area

**Fig. 1:**
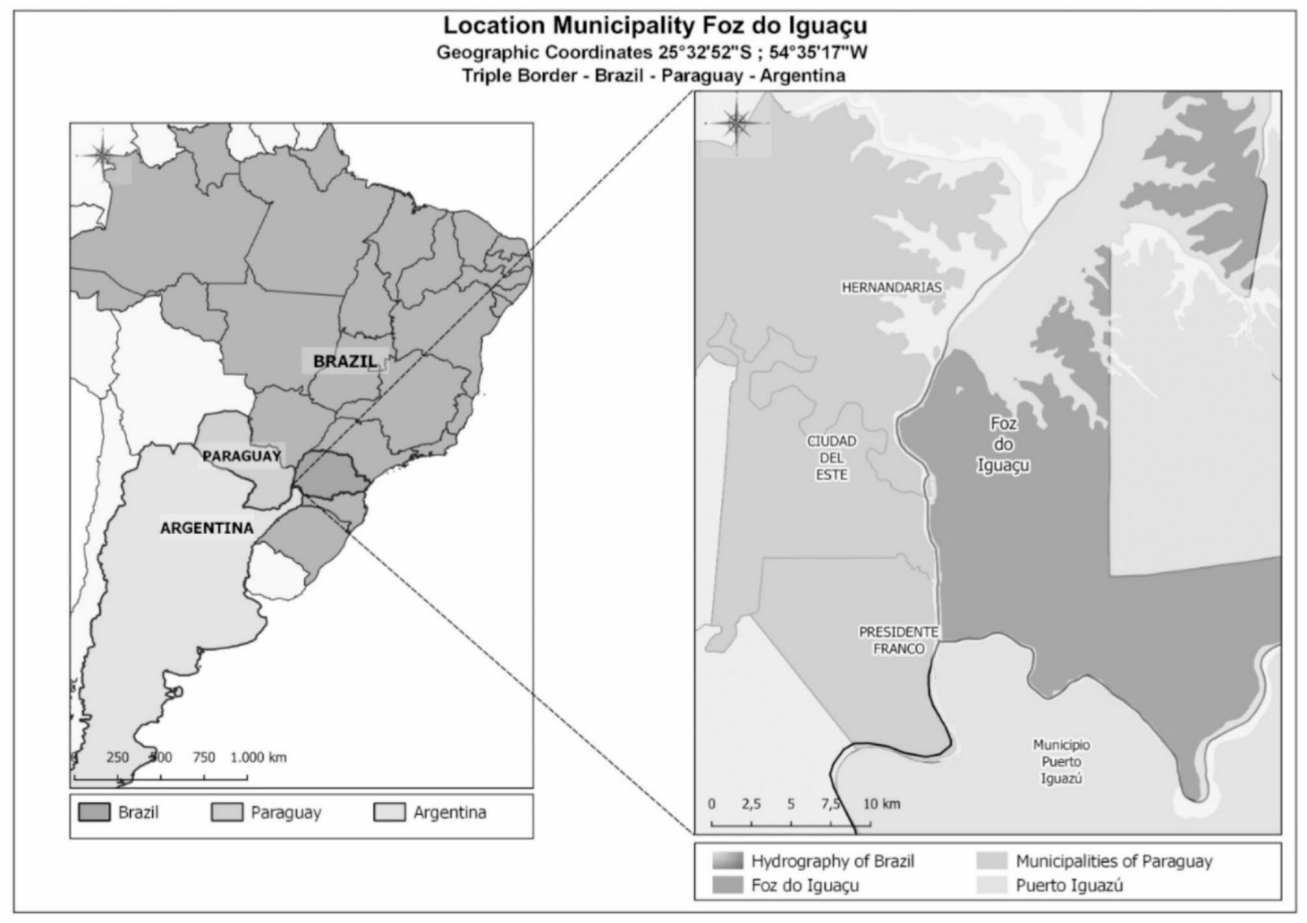
Mapping showing the location of Foz do Iguaçu and the triple border between Argentina, Brazil and Paraguay

Foz do Iguaçu (25°32’52"S; 54°35’17"W), with approximately 270,000 inhabitants, is situated in a triple international border region, sharing boundaries with Ciudad del Este, Hernandarias, and Presidente Franco in Paraguay, and Puerto Iguazu in Argentina (Fig. 1). The city lies in the Atlantic Forest biome, experiencing a humid subtropical mesothermal climate (Cfa) with an average annual rainfall of 1800 mm and mean temperatures ranging from 11 to 33 °C. Foz do Iguaçu boasts a high human development index, with an urbanization rate exceeding 99% and a population density of 414.58 inhabitants per km² [28]. Since the 1990s, the region, including Foz do Iguaçu, has been endemic for dengue. The city is divided into 73 areas, each containing an average of 1500 residential dwellings. In 2014, routine surveillance activities in Foz do Iguaçu adopted a One Health approach, integrating mosquito control, zoonoses, and venomous animals under a highly-trained field team [28].

### Fuzzy logic for predicting dengue transmission

The fuzzy inference system was employed to evaluate the spatiotemporal risk of DENV transmission in Foz de Iguaçu. Input parameters for the fuzzy system included epidemiological data (incidence of dengue cases), entomological data (the adult-based indicator TPI), climatic data (average soil temperature), and demographic data (demographic density). These indicators were considered within a spatiotemporal framework, with the spatial analysis scale based on the 73 areas of Foz do Iguaçu and the time scale covering January 2017 to December 2021. The calculation of the Receptivity for Dengue Transmission (Risk) utilized values recorded in the first fortnight of odd-numbered months (January, March, May, July, September, and November) for each year. Demographic Density data were derived from the 2010 Demographic Census.

### Entomological surveillance

Since January 2017, routine *Ae. aegypti* infestation surveys covered 100% of the urban area, involving the sampling of adult mosquitoes with traps. Roughly 3,500 Adultraps were distributed in urbanized areas, with one trap per 25 properties. All traps underwent inspection for four days during the first fortnight of every two-month period, resulting in approximately 105,000 trap inspections from 2017 to 2021. The entomological surveillance, focusing on adult mosquitoes, allowed for the development of entomological indicators, with the Trap Positivity Index (TPI) being a crucial factor in the Fuzzy model.

### Epidemiological surveillance

Foz do Iguaçu’s public health care and surveillance network includes 30 basic health units, 2 emergency care units, 3 private hospitals, and 1 public hospital. Dengue, Zika, and chikungunya are notifiable diseases recorded in a national information system (Sistema de Informação de Agravo de Notificação – SINAN). Suspected cases of dengue are identified based on disease notification, which is recorded in the SINAN system whenever patients present at least three symptoms within 15 days before seeking assistance, i.e., notifications are based on suspected rather than on confirmed cases. The incidence of dengue per area was estimated by dividing the total number of notified individuals by the corresponding human population, multiplied by 100,000.

### Demographic data

Demographic data for Foz do Iguaçu was sourced from the 2010 IBGE Demographic Census. The data from each of the 73 areas were grouped together, and human demographic density (DD) was calculated per area by dividing the total number of inhabitants by the size of the corresponding urban area (hab/km²).

### Soil temperature data

Mean soil temperature was obtained using the Google Earth Engine (GEE) platform, analyzing images from the Landsat 9 Level 2, Collection 2, Tier 2 dataset. The spatial resolution was 30 m, and temporal resolution was 16 days. The images were filtered using date and cloud cover criteria, and the soil temperature data for each urban area were extracted using QGIS^®^.

### Spatiotemporal distribution and geostatistical interpolated mapping

Traps and notified dengue cases were georeferenced using WGS 84. The spatial analysis utilized cartographic bases from IBGE and the Foz do Iguaçu Zoonosis Control Center. The spatial variability of dengue transmission risk was modeled by interpolating defuzzified values using the IDW (Inverse Distance Weighting) method. The Quantum GIS (QGIS^®^ version 3.10.2, https://www.qgis.org) was used for spatiotemporal analysis and thematic map construction.

### Spatial decision support system using fuzzy logic

**Fig. 2:**
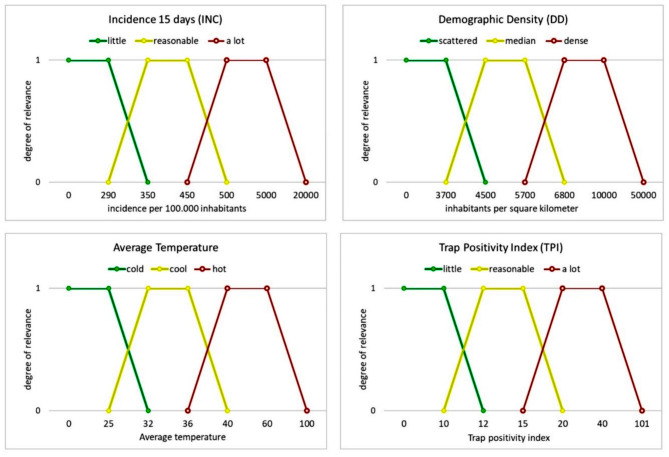
Membership function for the input variables: Incidence, Demographic Density, Average Temperature and Trap Positivity Index and their respective limits represented by pertinent functions trapezoid type

**Fig. 3:**
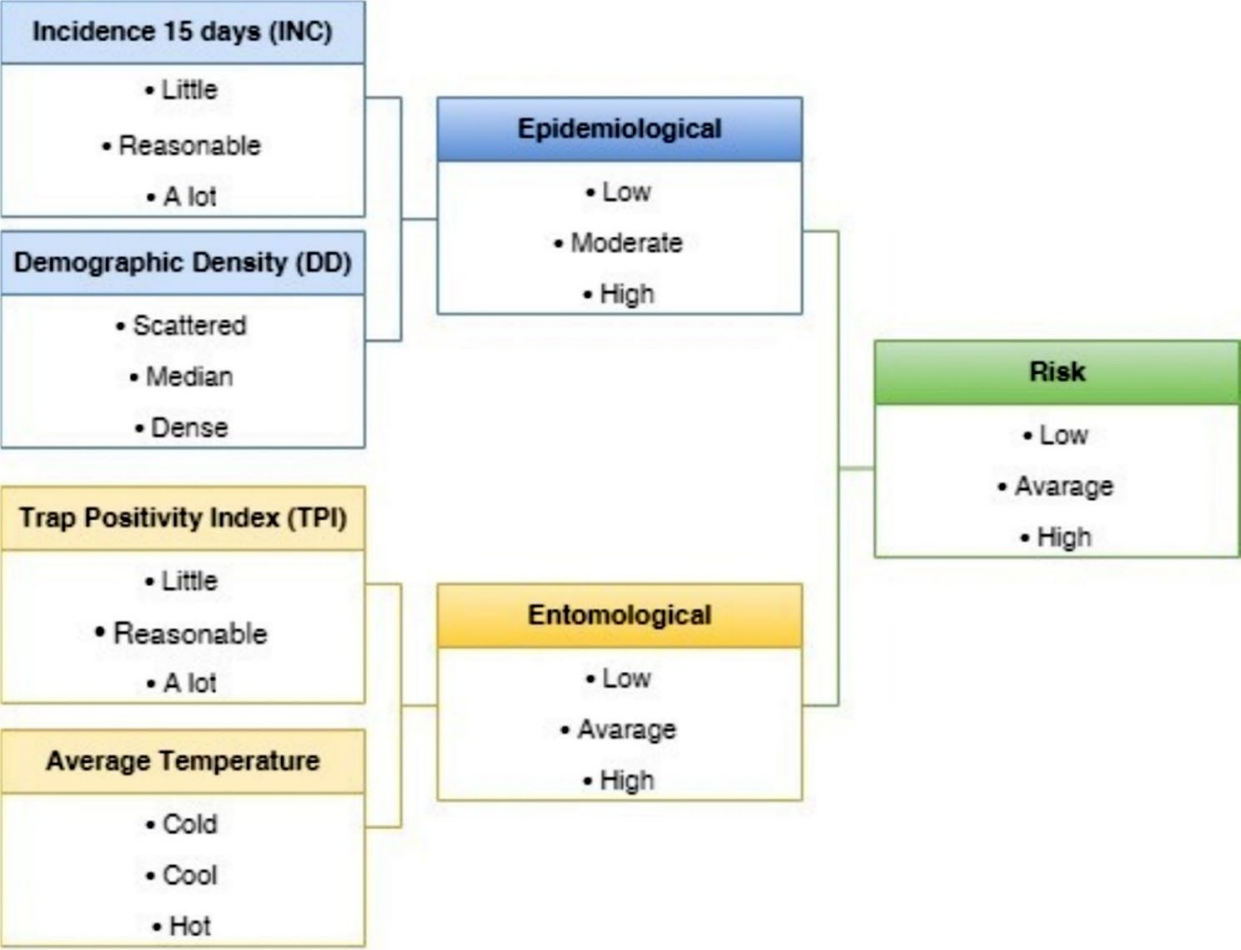
Fluxogram showing the combination of variables and their linguistic variables to evaluate the Fuzzy risk

A Mamdani-type fuzzy model, developed in Excel^®^ using VBA programming language, integrated arbovirus transmission risk indicators in Foz do Iguaçu (Fig. 2). Linguistic sets and pertinence values were defined for input and output fuzzy sets (Fig. 3). The fuzzy model’s rule base was formulated based on expert knowledge and literature data. Inputs were combined into pairs, generating Epidemiological and Entomological outputs, which were further combined into a final output called Risk [R].

A prospective model was adopted with a view to mapping areas of potential risk, as support for decision-making in the epidemiological surveillance of arboviruses. Fuzzy inference system was developed to integrate arbovirus transmission risk indicators in Foz do Iguaçu city. Variables related to the behavior and vector proliferation of *Ae. aegypti* were estimated based on linguistic sets, a rules base, and pertinence values were added for input and output fuzzy sets. To this end, a Mamdani-type fuzzy inference systems was developed and implemented in Excel^®^ software using VBA (Visual Basic for Applications) programming language. Initially, the input data was fuzzified in terms of linguistic sets (high, medium and low) and pertinence values between [0–1], where 0 indicates that it does not belong to the set, and 1 indicates that it fully belongs, were defined for each input and output fuzzy set (Supplementary Table 1). The input and output variables were represented by trapezoidal pertinence functions (Fig. 2). The fuzzy system rule base was formulated based on expert knowledge and data from the literature, using the following syntax: IF “antecedent” THEN “consequent”, considering the antecedent to be related to two or more “fuzzified” data using logical operators and the consequent to be the result of the risk calculation. The rule base is the core component of the fuzzy inference system, and the quality of its output largely depends on it. The fuzzy knowledge-based system, defined by the expert, consists primarily of fuzzy IF-THEN rules types. In this proposed system, a set of 27 types of fuzzy rules have been defined using the knowledge of the expert on the defined with the knowledge of the expert of the dengue transmission risk, based on the selected explanatory variables. The output data was defuzzified using the centroid technique and normalized into the interval [0-100]. The input variables were combined into pairs and subjected to a set of nine rules to generate the output. In this way, the inputs Incidence of Notified Suspected Dengue Cases (Inc) and Demographic Density (DD) generate the Epidemiological output and the inputs *Aedes aegypti* Mosquito Infestation Index (TPI) and Average Soil Temperature (t 0 C) generate the Entomological output. Epidemiological and Entomological were combined in new inputs, in a set of nine rules, generating the final output called Risk [R] (Fig. 3).

### Sensitivity analysis

The model incorporated expert knowledge through fuzzy set definitions, and a sensitivity analysis identified the contribution of each inference rule output. Rules that were not activated in the system were detected and removed.

The fuzzy system was tested with different datasets and adjusted the membership functions and rules to improve the accuracy and performance of the system. The formulation of fuzzy rules was based on knowledge of the problem domain and incorporated expert knowledge. Initially the rules for inferences were built combining all the rules. After this, the rules were presented to the group of experts to validate the decision support of the decision from system, related to the degree of relevance of the variables in fuzzy sets. Thus, the suggested changes were incorporated after each test. Additionally, a sensitivity evaluation process was performed to identify the contribution of each inference rule output and to detect and remove rules that have not been activated in the system. The advantage of this approach is the creation of sets of rules that produce decisions in a coherent way and that are fully justified by the databases from which the knowledge was extracted.

### Data analysis for fuzzy methodology

The fuzzy model was applied and validated with data from the 73 areas to identify priority areas for surveillance actions. The correct classification of areas as “High Risk” by the fuzzy methodology was evaluated by examining dengue incidence in these areas in the initial 15 days compared to the following 60-day interval. Additionally, a confusion matrix was generated comparing the classification of areas as High and the observed logarithmic incidence in the following 60-day interval, categorized if greater than the average of logarithmic incidence in the study period. The proportion of areas/periods predicting high risk and confidence intervals were computed using R platform [29], version 4.1.2, and plotted by areas and over time.

The fuzzy model was applied and validated with data from the 73 areas to identify priority areas for surveillance actions. Finally, a mapping was generated whose output is the identification of areas with different degrees of relevance for defining areas of potential dengue risk in the municipality. For evaluating the result of what was called correct classification of areas as “High Risk” areas by the Fuzzy methodology the fuzzy indicator pointed to a subset of pairs composed of city areas and time intervals evaluated at high risk for dengue outbreaks. Additionally, a confusion matrix was generated comparing the classification of areas as High and the observed logarithmic incidence in the following 60-day interval, categorized if greater than the average of logarithmic incidence in the study period. The proportion of areas/periods predicting high risk and confidence intervals were computed using R platform [29], version 4.1.2, and plotted by areas and over time.

**Fig. 4:**
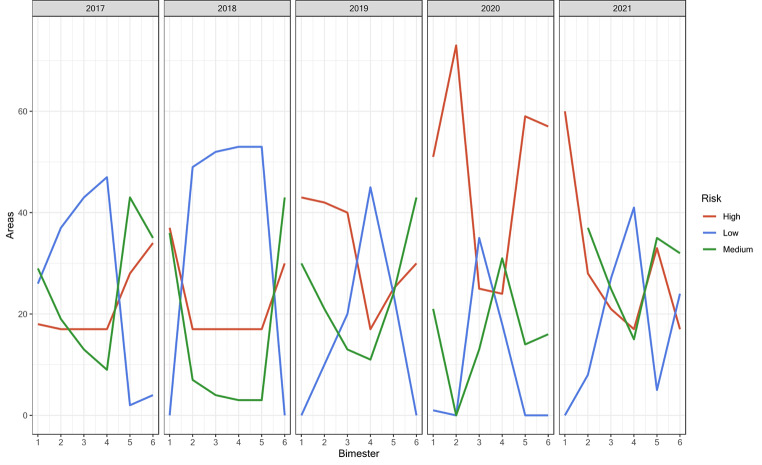
Number of Foz do Iguaçu city areas classified according to the risk level (low, medium, and high risk) per bimester from 2017 to 2021. The city has a total of 73 areas. The number of dengue cases reported per year within this timeframe was collected from the Sistema de Informação de Agravo de Notificação (SINAN)

The validation analysis was done by computing a proportion of the areas/periods in which the indicator predicts high risk of dengue, i.e., in hindsight a probability of success indicating the need for surveillance approaches in these areas. The proportion of success was analyzed in the study time and separately by areas or time. The confidence intervals were obtained from these samples using the Wilson score for Binomial proportions. Values obtained for the probability of success and confidence intervals were plotted by areas and over time. Analysis was done using R platform, version 4.1.2, and are shown in Fig. 4.

## Results

A sophisticated decision support system was devised, utilizing spatial and temporal information to aggregate environmental data through fuzzy rules. This system effectively detected priority territories and periods at risk of dengue, enabling strategic preventive intervention actions. In the municipality of Foz do Iguaçu between 2017 and 2021, diverse transmission scenarios associated with dengue risk were identified, providing a continuous risk surface highlighting areas with potential transmission conditions. This information is crucial for prioritizing specific strategic actions tailored to each area and facilitating the initiation of preventive measures. Over the 5-year period (2017 to 2021), a total of 438 areas were analyzed each year, resulting in 2190 analyses. This comprehensive spatial analysis was conducted using the dataset generated from the 73 areas for the spatial scale in Foz do Iguaçu (Fig. 4, Supplementary Table 2).

Of the total analyses conducted, 41.96% of the areas were classified as high risk, 29.54% as medium risk, and 28.49% as low risk. Notably, the annual accumulation of high-risk areas presented a tendency for increasing over time from 29.90% in 2017 to 40.18% in 2021, peaking 65.98% in 2020. Comparing the system’s predictions with the reported dengue cases revealed a correspondence, since the years with the highest number of areas classified as high risk (2019, 2020, and 2021) coincided with the years exhibiting the highest reported dengue cases. This observation underscores the system’s accuracy in identifying areas at risk, aligning with subsequent increases in dengue incidence during the evaluated period (Supplementary Table 2). The system effectively anticipated areas with heightened risk, contributing valuable insights for decision-makers at both spatial and temporal scales.

**Fig. 5:**
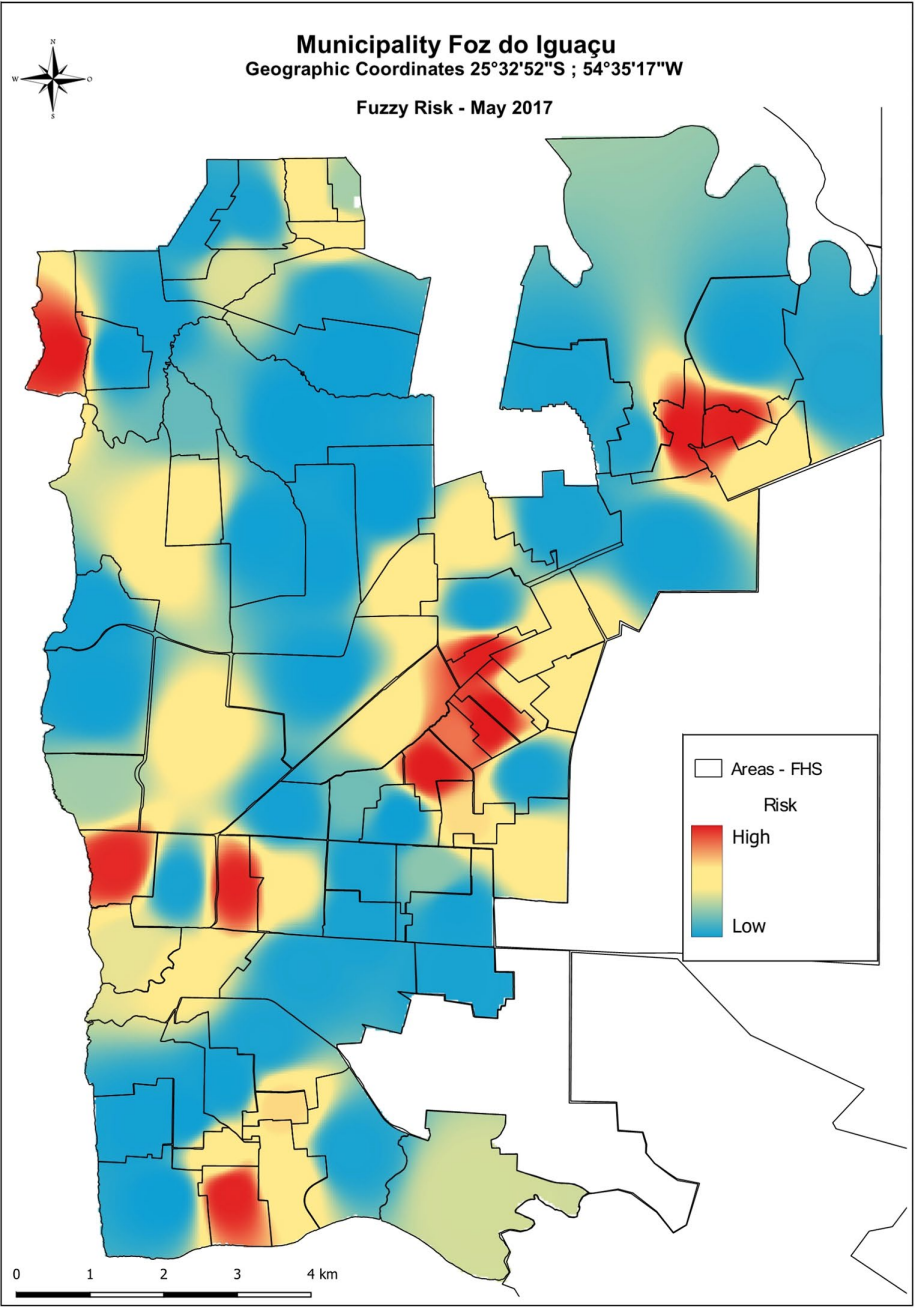
Definition of priority areas through the spatial decision support system for implementing specific environmental surveillance actions in the municipality of Foz do Iguaçu in May 2017, using fuzzy logic. The risk is represented by different colors in the map, with the blueish and reddish areas representing the low and high-risk areas, respectively

**Fig. 6:**
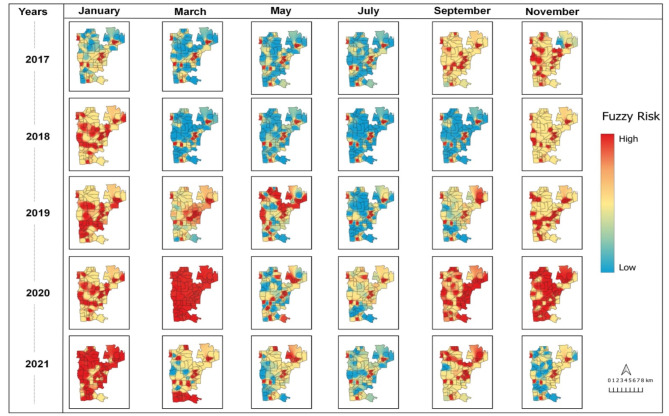
Spatiotemporal representation of priority areas defined by fuzzy risk for the implementation of specific environmental surveillance actions in the municipality of Foz do Iguaçu from 2017 to 2021

Starting from 2018, no areas were classified as low risk in the first and sixth bimesters, which were instead categorized as medium-high risk. The bimesters with the highest number of areas classified as high risk were the first (January and February) and the second (March and April). In contrast, the fourth bimester (July and August) had the lowest number of areas classified as high risk. Those findings respect the seasonal trend of both *Ae. aegypti* population density and dengue transmission patterns. The year 2020 witnessed an epidemic scenario characterized by a high transmission risk, with the majority of areas falling under the high-risk category. Among the evaluated time series, the system successfully pinpointed the third two-month period (May and June) as crucial for defining risk territories. This period marks the initiation of planning and implementation of local strategic actions, directing resources to combat dengue effectively. The Fig. 5 illustrates the risk assessment for the third two-month period, specifically focusing on May 2017. This map represents the ideal scenario for predicting environmental conditions conducive to dengue transmission, based on the defuzzified values obtained through the developed modeling. The goal is to expedite the implementation of control measures and predict dengue outbreaks efficiently. The integration of temporal and spatial information, as facilitated by the system, enhances the capacity to identify critical periods and areas at risk. This information empowers decision-makers to allocate resources effectively, enabling proactive measures to combat dengue and mitigate potential outbreaks. Consistent patterns were observed in the last two bimesters of 2020 and the first bimester of 2021, as outlined in Supplementary Tables 2 and illustrated in Fig. 6. Six bimonthly risk classification maps were generated for each year. While the areas classified as high risk varied between bimonths and across years, there was a discernible spatio-temporal dependence in 17 areas consistently classified as high risk. These areas exhibited a permanent high-risk profile (Fig. 6).

**Fig. 7:**
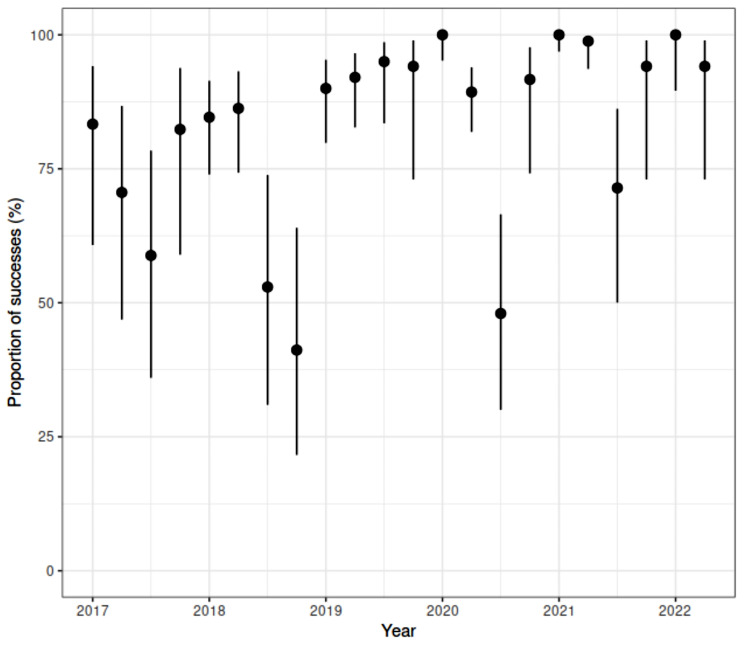
The probability of successful indication of high risk of dengue outbreaks in the City of Foz do Iguaçu from 2017 to 2022

**Fig. 8:**
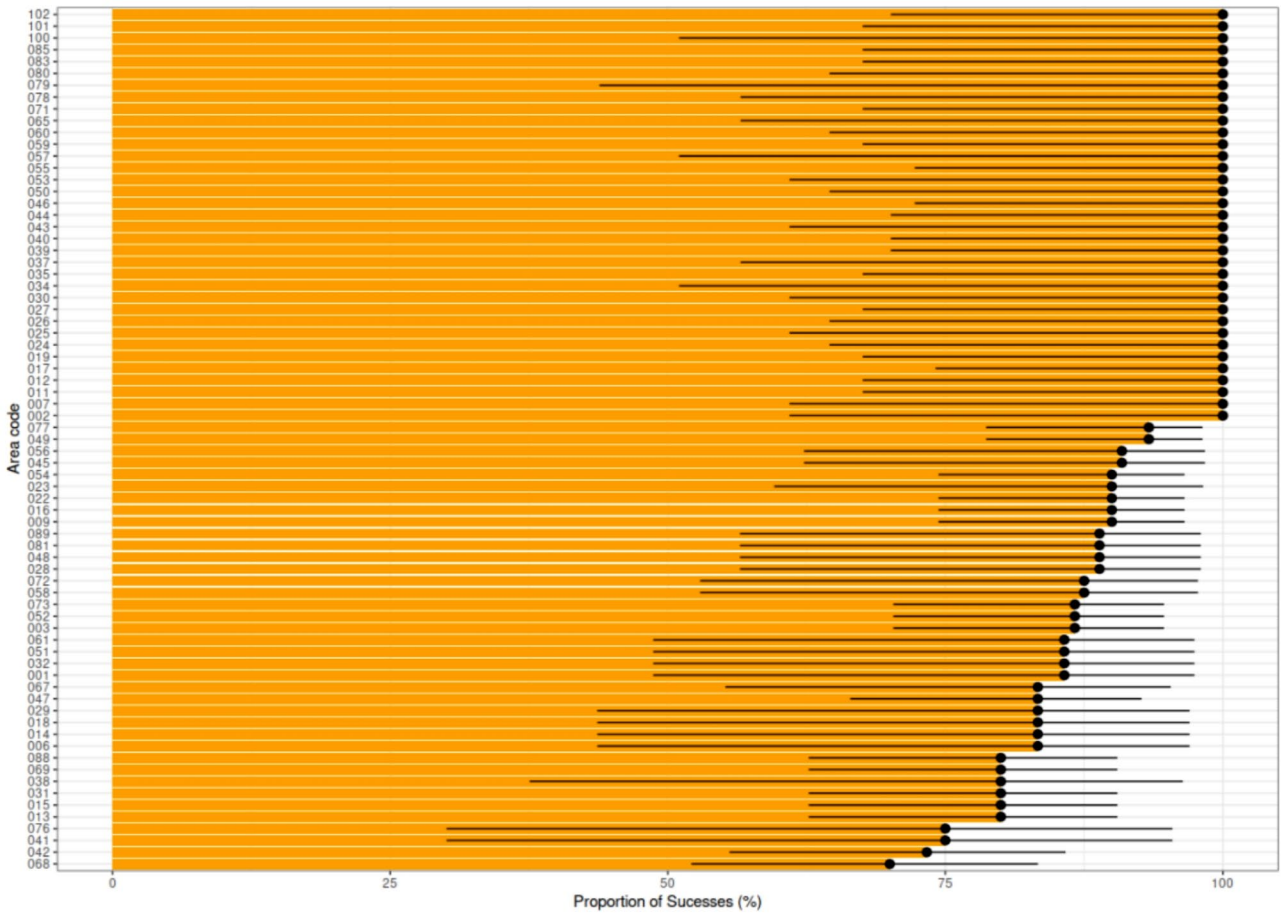
Proportion of successful indication of high-risk areas in the city of Foz do Iguaçu. Bars denote the mean values and black lines the 95% confidence intervals. Areas were ordered in decreasing order of mean values for the purpose of visualization

In the high-risk areas, there was a notable increase in the incidence of cases in the period following the risk measurement. Mean values for the probability of success were predominantly above 75%, indicating a strong correlation between high-risk classification and subsequent dengue incidence. A few time intervals exhibited proportions closer to 50%, notably at the end of 2018 and in the middle of 2020 (Fig. 7). The Fig. 8 underscores the high success rate (over 75%) for most areas in detecting an increase in dengue incidence within the first 15 days, based on the fuzzy indicator. Although a limited number of samples led to large 95% confidence intervals, these intervals predominantly concentrated above 50%, reinforcing the effectiveness of the fuzzy indicator in predicting dengue outbreaks.

The classification of areas as high risk had sensitivity value equal to 70%. The specificity was 49%. Together, this classification was statistically significant by p-value < 0.05. The cutoff value for the classification of observed incidences was 181 cases per 100,000 residents, as evaluated by the distribution of the logarithmic value of incidences. Also, for surveillance purposes a correct classification of high risk is more critical, thus signaling the importance of the sensitivity (Supplementary Table 3). The observed temporal patterns and spatio-temporal dependence in high-risk areas provide valuable insights for targeted intervention strategies. The consistent success rates in predicting dengue incidence affirm the reliability of the fuzzy indicator, emphasizing its potential as a proactive tool for early detection and timely response to potential outbreaks.

## Discussion

The escalating transmission of arboviruses worldwide [30] underscores the imperative need for the development of methods capable of comprehensively analyzing and translating the risk of these globally significant diseases in a qualitative, quantitative, and spatiotemporal context [9, 14]. The transmission dynamics of arboviruses exhibit a distinct seasonal pattern, influenced by various biotic and abiotic factors acting along a spatiotemporal axis. Moreover, the heterogeneous nature of urban spaces within large metropolises and cities, as highlighted by Dzul-Manzanilla, emphasizes the necessity to comprehend local transmission dynamics for subsequent risk area characterization [31]. Presently, numerous mathematical models have been devised to enhance arbovirus surveillance techniques, addressing this formidable public health challenge [30, 32]. Encouragingly, initiatives supporting the development and application of these models should be increasingly promoted. Nevertheless, a substantial gap persists between model development and their effective translation into robust surveillance practices. In this manuscript, we used the Fuzzy logic to determine the areas under higher dengue transmission risk in Foz do Iguaçu and evaluated this system as a decision-making tool to prioritize areas for further vector control interventions.

The high-risk areas for dengue transmission in Foz do Iguaçu shares some common characteristics. These high-risk areas have similarities in terms of dense land occupation, small size housing and high human population densities with low per capita income. Additionally, entomological indicators based on adult mosquito sampling are higher in those areas than compared to other sections of the city, and more often heat islands were found through temperature satellite monitoring. This information alone characterizes the heterogeneity of the municipality’s territory in a practical and objective way - the risk areas where outbreaks occur and trigger epidemics, and where prevention and control actions should be carried out as a priority.

Biotic and abiotic factors play crucial roles in shaping the dynamics of arbovirus transmission, either at the global or local level. Since this manuscript aims to determine the hotspots of arbovirus transmission using entomological, demographic, environmental and epidemiological data gathered at a city level, we will limit discussing the effects of biotic and abiotic factors locally. The *Ae. aegypti* mosquito is collected in higher densities at the more urbanized areas because it is highly adapted to live in close association with human dwellings. *Aedes aegypti* females preferentially blood feed on humans rather than in other domesticated hosts, lay their eggs on artificial breeding sites with preference for large, shaded containers located in the peridomestic area [1, 33–38]. This species has a limited flight range, dispersing no further than a few hundred meters from their breeding site [4, 39, 40]. Conversely, mosquito distribution over endemic cities tend to overlap with human demography, i.e., higher vector densities are expected to be found in the regions with higher human population [41, 42]. Tropical cities in developing countries have heterogeneous landscape, socioeconomic conditions and infrastructure, favorable environment, resulting in a heterogeneous risk of arbovirus transmission [10, 11, 31, 43]. This heterogeneity is the outcome of several factors such as piped water availability, human behavior regarding water storage, community engagement in dengue good practices, and waste management disposal to avoid them becoming breeding sites for mosquito females [44–46]. Such observation highlights how complex the dengue transmission dynamics can be in urban settings, eliciting therefore a multifactorial approach to mitigate its burden. In that sense, the fuzzy logic is presented as an alternative strategy to improve vector surveillance.

Fuzzy logic is an approach to variable processing that enables the handling of multiple possible truth values within the same variable. It aims to address problems characterized by an open, imprecise spectrum of data, employing heuristics to derive a range of accurate conclusions. The Fuzzy logic was used at least in two distinct opportunities to investigate dengue transmission dynamics in South America. Epidemiological data as dengue and severe dengue cases recorded between 1997 and 2011 in Colombia was used to forecast disease occurrence between 2012 and 2015 with greater efficacy than traditional models [47]. A broader investigation applied fuzzy logic to assess the biogeographical risk of dengue in South America and implied *Ae. aegypti* distribution as the primary vector of dengue in the continent [48]. Although the role of *Ae. aegypti* as dengue vector is well-known, their fuzzy system used environmental and demographic variables on its assessment [48]. We used the fuzzy logic to model locally acquired entomological, demographic, environmental, and epidemiological data to estimate the output variables, i.e., transmission risk. Importantly, we interpolated defuzzified values to translate fuzzy logic into a map of the city, pinpointing geographic areas with higher dengue transmission risk. Given the challenges in maintaining effective surveillance and efficient vector control interventions across the entire extent of a city, identifying the spatiotemporal occurrence of high-risk areas becomes crucial for directing targeted vector control activities, including ULV fogging and removing breeding sites. This remains one of the significant challenges faced by public health stakeholders involved in arbovirus transmission.

In developing our surveillance model and practical decision-making process based on evidence using fuzzy logic, we carefully identified and utilized relevant data for arbovirus surveillance. This data, readily available to managers of arbovirus prevention and control programs, is accessible for free or can be produced and organized in a straightforward and practical manner, seamlessly integrating into service routines. The selected data, considered easy to acquire and collected from official sources, includes: (i) incidence of cases; (ii) demographic density; (iii) freely available satellite data on soil temperature in urban areas; and (iv) entomological data produced in the routine vector prevention and control service through adult Aedes capture traps [28, 49]. Applying fuzzy logic to this data allowed us to generate information on the risk of arbovirus transmission. While employing different techniques, our results effectively highlighted the heterogeneity of arbovirus transmission risk in urban environments, aligning with findings observed elsewhere [31, 50–53]. The fuzzy method enabled the identification of areas with a permanent receptivity to dengue epidemics. In the analyzed reality, the municipality’s heterogeneity was evident, revealing that out of 73 areas, 17 are at a high permanent risk for dengue transmission. This emphasizes the need for intense and continuous specific environmental surveillance strategies for prevention and control, emphasizing the necessity for differentiated actions based on risk and epidemiological timing.

We also assessed the model’s capability to signal and anticipate the risk of an increase in dengue cases. A practical example of utilizing the obtained results involves condensing the outcomes of high-risk areas across all years by bimester, revealing marked seasonality. Bimesters 1, 2, 5, and 6 demonstrated the highest numbers of areas in high-risk conditions, while bimesters 3 and 4 had the lowest. This strategic insight informs the design and implementation of prevention and control strategies, preventing the impact of high-risk areas observed in the third and fourth bimesters on surrounding areas, thus mitigating transmission amplification. Months from May to August, historically characterized by lower incidence, infestation rates, and temperatures, present a prime opportunity to intensify actions aimed at blocking transmission. With fewer areas classified as high risk during these months, there is a significant opportunity to interrupt arbovirus transmission in these zones and prevent transmission amplification in their surroundings.

Although we believe our manuscript produces a relevant and robust map that defines the priority areas for further vector control interventions, it is important to address the limitations in our analysis and datasets. Fuzzy logic is a robust method for managing uncertainties and variability in complex systems, as demonstrated herein. However, its application presents several challenges. The development of fuzzy sets and their membership functions can be intricate and subjective. Therefore, an inappropriate selection of these sets can result in inaccurate outcomes. Effectively modeling a fuzzy system necessitates specific expertise regarding the problem, its variables, and their interactions. Inaccurate data or misinterpretation of primary data can undermine the model’s reliability. Post-development, the fuzzy system requires calibration to align with real-world data, a process that can be both complex and time-consuming that requires reliable data. Additionally, fuzzy logic may struggle with rapidly evolving data, such as those seen in dengue outbreaks, necessitating continuous model adjustments. Consequently, we should be aware that fuzzy logic produced a robust endpoint analysis using this specific dataset comprised from Jan 2017 to Dec 2021. New fuzzy models should be developed using additional data from Foz do Iguaçu or data from somewhere else due to its specificity, which means we should be careful about extrapolating our findings to other dengue endemic sites.

## Conclusions

Our study demonstrated that the integration of entomological, environmental, demographic, and epidemiological data, along with the collaborative exchange of insights between local public health professionals and scientists, significantly improved the identification of risk areas, action planning, and support for evidence-based decision-making. The application of the Fuzzy Methodology facilitated the modeling of complex scenarios through a systematic process. Key advantages of employing fuzzy logic include (a) leveraging the technical and practical expertise of vector control professionals; (b) utilizing freely available data sources; (c) accessing simple and user-friendly software for efficient data collection and subsequent analysis; and (d) providing a standardized methodology for analysis. The active involvement of service technicians with substantial expertise, along with the importance of their technical knowledge in parameterizing variables, played a crucial role in the success of this research [28]. Fuzzy logic demonstrated its capability to characterize risk in the territory over time and guide timely and location-specific decision-making by analyzing and characterizing risks through the collective analysis of multiple variables.

## Electronic supplementary material

Below is the link to the electronic supplementary material.

Supplementary Material 1

## Data Availability

The datasets used and/or analysed during the current study are available from the corresponding author on reasonable request.

## Abbreviations

DENV: Dengue virus
ZIKV: Zika virus
CHIKV: Chikungunya virus
TPI: Trap Positivity Index
DD: Demographic Density
GEE: Google Earth Engine
IDW: Inverse Distance Weighting
VBA: Visual Basic for Applications
ULV: Ultra-Low Volume
