# Supplementary material for: The fuzzy system ensembles entomological, epidemiological, demographic and environmental data to unravel the dengue transmission risk in an endemic city
Source: BMC Public Health. 2024 Sep 27;24:2587. doi: 10.1186/s12889-024-19942-4 (PMC11430332; doi:10.1186/s12889-024-19942-4)
Supplement: Supplementary file 1 — Supplementary Material 1 [file 12889_2024_19942_MOESM1_ESM.docx]

**Supplementary Table 1:** The fuzzy rules base that has been used to define the nine fuzzy rules defined with the knowledge of the expert in the domain of the dengue transmission risk.

| **Rule 1** | ***IF*** | Incidence is *Little* | ***AND*** | Demographic Density is *Scattered* | ***THEN*** | Epidemiological is *Low* |
| --- | --- | --- | --- | --- | --- | --- |
| **Rule 2** | ***IF*** | Incidence is *Little* | ***AND*** | Demographic Density is *Median* | ***THEN*** | Epidemiological is *Avarage* |
| **Rule 3** | ***IF*** | Incidence is *Little* | ***AND*** | Demographic Density is *Dense* | ***THEN*** | Epidemiological is *High* |
| **Rule 4** | ***IF*** | Incidence is *Reasonable* | ***AND*** | Demographic Density is *Scattered* | ***THEN*** | Epidemiological is *Avarage* |
| **Rule 5** | ***IF*** | Incidence is *Reasonable* | ***AND*** | Demographic Density is *Median* | ***THEN*** | Epidemiological is *Avarage* |
| **Rule 6** | ***IF*** | Incidence is *Reasonable* | ***AND*** | Demographic Density is *Dense* | ***THEN*** | Epidemiological is *High* |
| **Rule 7** | ***IF*** | Incidence is *A Lot* | ***AND*** | Demographic Density is *Scattered* | ***THEN*** | Epidemiological is *High* |
| **Rule 8** | ***IF*** | Incidence is *A Lot* | ***AND*** | Demographic Density is *Median* | ***THEN*** | Epidemiological is *High* |
| **Rule 9** | ***IF*** | Incidence is *A Lot* | ***AND*** | Demographic Density is *Dense* | ***THEN*** | Epidemiological is *High* |
| **Rule 10** | ***IF*** | Trap Positivity Index is *Little* | ***AND*** | Average Temperature is *cold* | ***THEN*** | Entomological is *Low* |
| **Rule 11** | ***IF*** | Trap Positivity Index is *Little* | ***AND*** | Average Temperature is *Cool* | ***THEN*** | Entomological is *Low* |
| **Rule 12** | ***IF*** | Trap Positivity Index is *Little* | ***AND*** | Average Temperature is *Hot* | ***THEN*** | Entomological is *Avarage* |
| **Rule 13** | ***IF*** | Trap Positivity Index is *Reasonable* | ***AND*** | Average Temperature is *cold* | ***THEN*** | Entomological is *Low* |
| **Rule 14** | ***IF*** | Trap Positivity Index is *Reasonable* | ***AND*** | Average Temperature is *Cool* | ***THEN*** | Entomological is *Avarage* |
| **Rule 15** | ***IF*** | Trap Positivity Index is *Reasonable* | ***AND*** | Average Temperature is *Hot* | ***THEN*** | Entomological is *High* |
| **Rule 16** | ***IF*** | Trap Positivity Index is *A Lot* | ***AND*** | Average Temperature is *cold* | ***THEN*** | Entomological is *Avarage* |
| **Rule 17** | ***IF*** | Trap Positivity Index is *A Lot* | ***AND*** | Average Temperature is *Cool* | ***THEN*** | Entomological is *Avarage* |
| **Rule 18** | ***IF*** | Trap Positivity Index is *A Lot* | ***AND*** | Average Temperature is *Hot* | ***THEN*** | Entomological is *High* |
| **Rule 19** | ***IF*** | Epidemiological is *Low* | ***AND*** | Entomological is *Low* | ***THEN*** | Risk is *Low* |
| **Rule 20** | ***IF*** | Epidemiological is *Low* | ***AND*** | Entomological is *Avarage* | ***THEN*** | Risk is *Avarage* |
| **Rule 21** | ***IF*** | Epidemiological is *Low* | ***AND*** | Entomological is *High* | ***THEN*** | Risk is *High* |
| **Rule 22** | ***IF*** | Epidemiological is *Moderate* | ***AND*** | Entomological is *Low* | ***THEN*** | Risk is *Low* |
| **Rule 23** | ***IF*** | Epidemiological is *Moderate* | ***AND*** | Entomological is *Avarage* | ***THEN*** | Risk is *Avarage* |
| **Rule 24** | ***IF*** | Epidemiological is *Moderate* | ***AND*** | Entomological is *High* | ***THEN*** | Risk is *High* |
| **Rule 25** | ***IF*** | Epidemiological is *High* | ***AND*** | Entomological is *Low* | ***THEN*** | Risk is *High* |
| **Rule 26** | ***IF*** | Epidemiological is *High* | ***AND*** | Entomological is *Avarage* | ***THEN*** | Risk is *High* |
| **Rule 27** | ***IF*** | Epidemiological is *High* | ***AND*** | Entomological is *High* | ***THEN*** | Risk is *High* |

**Supplementary Table 2:** Number of Foz do Iguaçu city areas classified according to the risk level (low, medium, and high risk) per bimester from 2017 to 2021. The city has a total of 73 areas. The number of dengue cases reported per year within this timeframe was collected from the Sistema de Informação de Agravo de Notificação (SINAN).

| **Year**  **(dengue cases)** | **Bimester** | **Low Risk** | **Medium Risk** | **High Risk** |
| --- | --- | --- | --- | --- |
| **2017**  (1,923) | 1 | 26 | 29 | 18 |
|  | 2 | 37 | 19 | 17 |
|  | 3 | 43 | 13 | 17 |
|  | 4 | 47 | 9 | 17 |
|  | 5 | 2 | 43 | 28 |
|  | 6 | 4 | 35 | 34 |
| **2018**  (1,958) | 1 | - | 36 | 37 |
|  | 2 | 49 | 7 | 17 |
|  | 3 | 52 | 4 | 17 |
|  | 4 | 53 | 3 | 17 |
|  | 5 | 53 | 3 | 17 |
|  | 6 | - | 52 | 21 |
| **2019**  (11,625) | 1 | - | 30 | 43 |
|  | 2 | 10 | 21 | 42 |
|  | 3 | 20 | 13 | 40 |
|  | 4 | 45 | 11 | 17 |
|  | 5 | 24 | 24 | 25 |
|  | 6 | - | 43 | 30 |
| **2020**  (27,427) | 1 | 1 | 21 | 51 |
|  | 2 | - | - | 73 |
|  | 3 | 35 | 13 | 25 |
|  | 4 | 18 | 31 | 24 |
|  | 5 | - | 14 | 59 |
|  | 6 | - | 16 | 57 |
| **2021**  (10,752) | 1 | - | 13 | 60 |
|  | 2 | 8 | 37 | 28 |
|  | 3 | 27 | 25 | 21 |
|  | 4 | 41 | 15 | 17 |
|  | 5 | 5 | 35 | 33 |
|  | 6 | 24 | 32 | 17 |
| **Total** | | **624** | **647** | **919** |

**Supplementary Table 3:** Confusion matrix of classification of high-risk pairs of areas/bimester using the fuzzy approach compared to categorization of incidence in the 60-day following period.

|  | **Incidence greater than average** | | |
| --- | --- | --- | --- |
| **Risk Classification** | Low | High |  |
| Low | 3902 | 3637 |  |
| High | 1661 | 3550 |  |
| Sensitivity | 70% |  |  |
| Specificity | 49% |  |  |
